# Aging-dependent skin microbiome alterations across body sites in a United Kingdom cohort

**DOI:** 10.3389/fragi.2025.1644012

**Published:** 2025-09-19

**Authors:** Mary Hannah Swaney, Duncan J. Newman, Junhong Mao, Anthony C. Hilton, Tony Worthington, Min Li

**Affiliations:** ^1^ Colgate-Palmolive Company, Piscataway, NJ, United States; ^2^ College of Health and Life Sciences, Aston University, Birmingham, United Kingdom

**Keywords:** skin microbiome, aging skin, aging skin microbiome, microbial diversity, skin

## Abstract

**Introduction:**

The aging process profoundly influences not only the health and visual appearance of the skin, but also the composition of the microbial communities residing on its surface.

**Methods:**

To investigate these microbial changes, we employed a comprehensive, multi-scale approach that probes community composition, species interactions, and predicted metabolic function of the skin microbiome of the face and forearm in young and old age individuals from the United Kingdom using 16S rRNA gene sequencing.

**Results:**

Our findings revealed significant and site-specific age-related shifts in the microbiome involving diversity, interpersonal heterogeneity, network connectivity, and metabolic potential, suggesting loss of microbiome robustness and a shift towards a hyperdiversified, fragile microbial community in old age. Furthermore, we applied Dirichlet Multinomial Mixtures to uncover novel age-driven microbiome profiles unique across each skin site, highlighting *Cutibacterium acnes*, *Staphylococcus hominis*, and microbial community diversity as key differentiating biomarkers of the skin microbiome across the lifespan.

**Discussion:**

Overall, through examining the aging skin microbiome from a systems perspective, our study reinforces and enhances the findings from previous aging microbiome studies and underscores the importance of site-specific differences in skin microbiome dynamics with age. These insights suggest that microbial interventions could mitigate age-related changes, enhancing skin health and wellbeing throughout life.

## Introduction

The human skin microbiome is the collection of microorganisms that inhabits the skin and carries out beneficial and necessary processes to support skin health (Byrd et al., 2018). Starting at birth, the skin microbiota play a critical role in the development and homeostatic maintenance of the immune system and skin physiology over one’s lifetime. For example, they influence key skin functions that include acidification, antimicrobial defense, lipid synthesis, and barrier integrity (Byrd et al., 2018; Almoughrabie et al., 2023; Zheng et al., 2022; Liu et al., 2023). Balance of the skin microbiome is crucial; an imbalance, or dysbiosis, can disrupt these functions and is associated with various conditions including atopic dermatitis and acne vulgaris (Nakatsuji and Gallo, 2018; Huang et al., 2023). Therefore, supporting the host-microbiome interactions at the skin surface is critical for maintaining the delicate balance between health and dermatological conditions.

Aging is a time-dependent process characterized by a decline in functional capacity that affects nearly all living organisms (López-Otín et al., 2013; Guo et al., 2022). It involves not only a decline in tissue and cellular function, but also broader deterioration of physiological processes, which can lead to increased inflammation and visible alterations in appearance, particularly in the skin (López-Otín et al., 2023; Tobin, 2017). The skin experiences significant transformations during aging due to intrinsic factors like reduced epidermal thickness, slower cell turnover, decreased collagen production, and changes in immune function, as well as extrinsic factors such as sun exposure, pollutants, and smoking (Farage et al., 2008). These factors collectively alter the physiological properties of the skin, leading to variations in pH, lipid and amino acid composition, and reduced sweat and sebum production (Choi, 2019; Schreiner et al., 2000; Waller and Maibach, 2006). Such differences affect the skin microbiota, which rely on the skin as a microbial niche and function to support a healthy skin barrier.

Aging skin undergoes significant alterations in its microbiome compared to that of younger individuals, with the skin microbiome reported to be an accurate predictor of chronological age, more so than the gut or oral microbiomes (Huang et al., 2020). With age, there is a notable rise in alpha diversity, which often coincides with a reduction in *Cutibacterium* (Shibagaki et al., 2017; Jugé et al., 2018; Howard et al., 2022; Ying et al., 2015; Kim et al., 2022; Larson et al., 2022). This shift is further marked by a rise in *Corynebacterium* and *Streptococcus* populations (Shibagaki et al., 2017; Jugé et al., 2018; Howard et al., 2022). The specific bacteria at the species level that differentiate young and aged skin can vary significantly from study to study, reflecting the substantial impact of population demographics, environmental factors, and methodological differences on the identification of age-related species of interest (Kim et al., 2022; Larson et al., 2022; Myers et al., 2023; Garlet et al., 2024). Despite this variability, numerous studies consistently identify *Cutibacterium acnes* and *Corynebacterium kroppenstedtii* as key markers of age-related microbiome changes (Larson et al., 2022; Garlet et al., 2024; Dimitriu et al., 2019; Zhou et al., 2023). *C. acnes* tends to decrease with age, while *C. kroppenstedtii* increases, underscoring their roles as key indicators of microbiome shifts that could influence skin health and the aging process. Furthermore, the skin microbiome is generally stable and resilient to environmental exposures during adulthood (Oh et al., 2016). However, in old age, there is an observed increase in interpersonal variation in the skin microbiome, indicating decline of microbiome stability and robustness (Jugé et al., 2018; Howard et al., 2022; Ying et al., 2015; Larson et al., 2022; Garlet et al., 2024).

While many studies have thoroughly evaluated the microbiome of the face as it ages (Huang et al., 2020; Shibagaki et al., 2017; Jugé et al., 2018; Howard et al., 2022; Kim et al., 2022; Larson et al., 2022; Myers et al., 2023; Garlet et al., 2024; Dimitriu et al., 2019; Zhou et al., 2023), there is a gap in comprehensive understanding of the aging microbiome of non-facial skin sites. Characterizing the aging skin microbiome across the body is crucial for uncovering insights into susceptibility to infections, skin conditions, and overall skin health. Moreover, as consumer interest in the “skinification” of beauty and personal care products grows, there is a demand for targeted solutions that address diverse aspects of skin health, including anti-aging treatments and personalized care. As the skin microbiome is a pivotal component of skin health, evaluating the microbiome variations with age across different body sites is essential. This broader understanding presents a unique opportunity for developing innovative interventions and products that can enhance skin vitality and wellbeing across the lifespan. Expanding this research not only contributes to a more holistic understanding of aging and skin health but also underscores the potential for microbiome-focused interventions to mitigate age-related skin changes.

In this study, we characterized the skin microbiome of the face and forearm in young and old aged individuals from a UK-based population using 16S rRNA gene sequencing. The objective of our study was to evaluate the differences in composition, species interactions, and predicted metabolic function of the bacterial community with age across two distinct skin sites. By focusing on a UK-based population, we aimed to provide insights into aging-related skin microbiome differences of this geographical region, which is relatively understudied compared to North America- and Asia-based populations. We employed comprehensive techniques including differential abundance testing, microbial association network analysis, microbiome clustering, and functional prediction to identify age-related patterns in the skin microbiome and to enhance our understanding of the skin microbiome’s interplay with the aging process.

## Results

A total of 59 participants from Birmingham, United Kingdom were enrolled in the study, including thirty young age (YA) (26.7 ± 4.45 years) and twenty-nine old age (OA) (72.3 ± 4.04 years) individuals (Supplementary Table S1). The gender distribution was similar for both age groups (YA: n = 12 male and n = 18 female; OA: n = 12 male and n = 17 female). Other participant demographics and clinical characteristics are summarized in Supplementary Table S1. Skin microbiome samples were collected from the antecubital fossa (arm) and cheek (face) of the participants and were sequenced using V1-V3 16S rRNA gene sequencing, yielding a median of 105,646 high-quality sequences per sample across both sites.

### Microbiome diversity and composition vary by age and skin site

**FIGURE 1:**
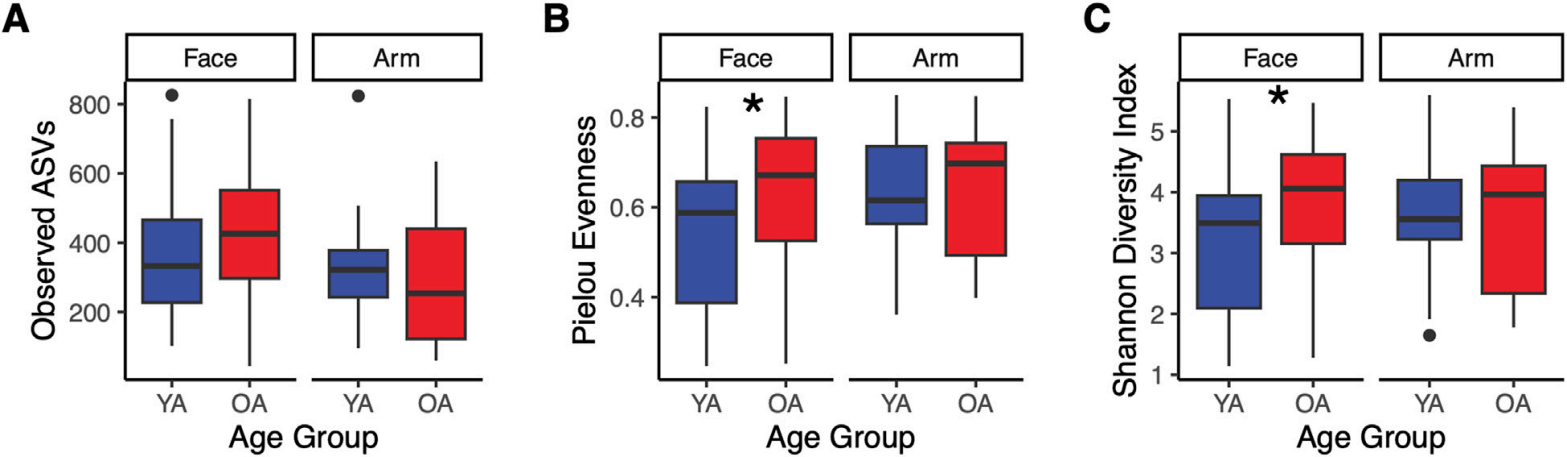
Increased alpha diversity in the face microbiome with age. Alpha diversity metrics were calculated and compared between the old age (OA) group and young age (YA) group for the face and arm. Calculations were performed on the ASV feature table and include: **(A)** Observed ASVs, **(B)** Pielou evenness, and **(C)** Shannon diversity. YA (blue) represents the young age group, and OA (red) represents the old age group. Points represent data outliers. Statistical significance was calculated using the Wilcoxon rank sum test (* *p*-value <0.05).

Alpha diversity metrics Shannon diversity and Pielou evenness were significantly higher in the old age group compared to the young age group on the face (*p* = 0.043 and *p* = 0.023, respectively), with no significant differences in richness (number of observed amplicon sequence variants (ASVs)) (Figure 1). This indicates that the increase in diversity is due to a more even microbial community rather than a greater number of taxa. In contrast, no significant differences in richness, evenness, or Shannon diversity were observed on the arm with age (Figure 1), highlighting that the general pattern of increased alpha diversity of the skin microbiome with age may be skin site-specific. Indeed, the majority of skin microbiome studies reporting significant increases in alpha diversity with age observed these differences on the face (Shibagaki et al., 2017; Jugé et al., 2018; Kim et al., 2022; Myers et al., 2023; Garlet et al., 2024; Li et al., 2020; Sun et al., 2024; Kim et al., 2019). We also assessed differences in alpha diversity attributed to gender. For the arm, there were no significant age-related differences for either gender, similar to the findings for the overall cohort (Supplementary Figures S1A–C). Conversely, for the face, only female participants exhibited a significant increase in Shannon diversity and Pielous evenness, indicating that gender may be a driving factor for the observed facial microbial diversity increase with age.

**FIGURE 2:**
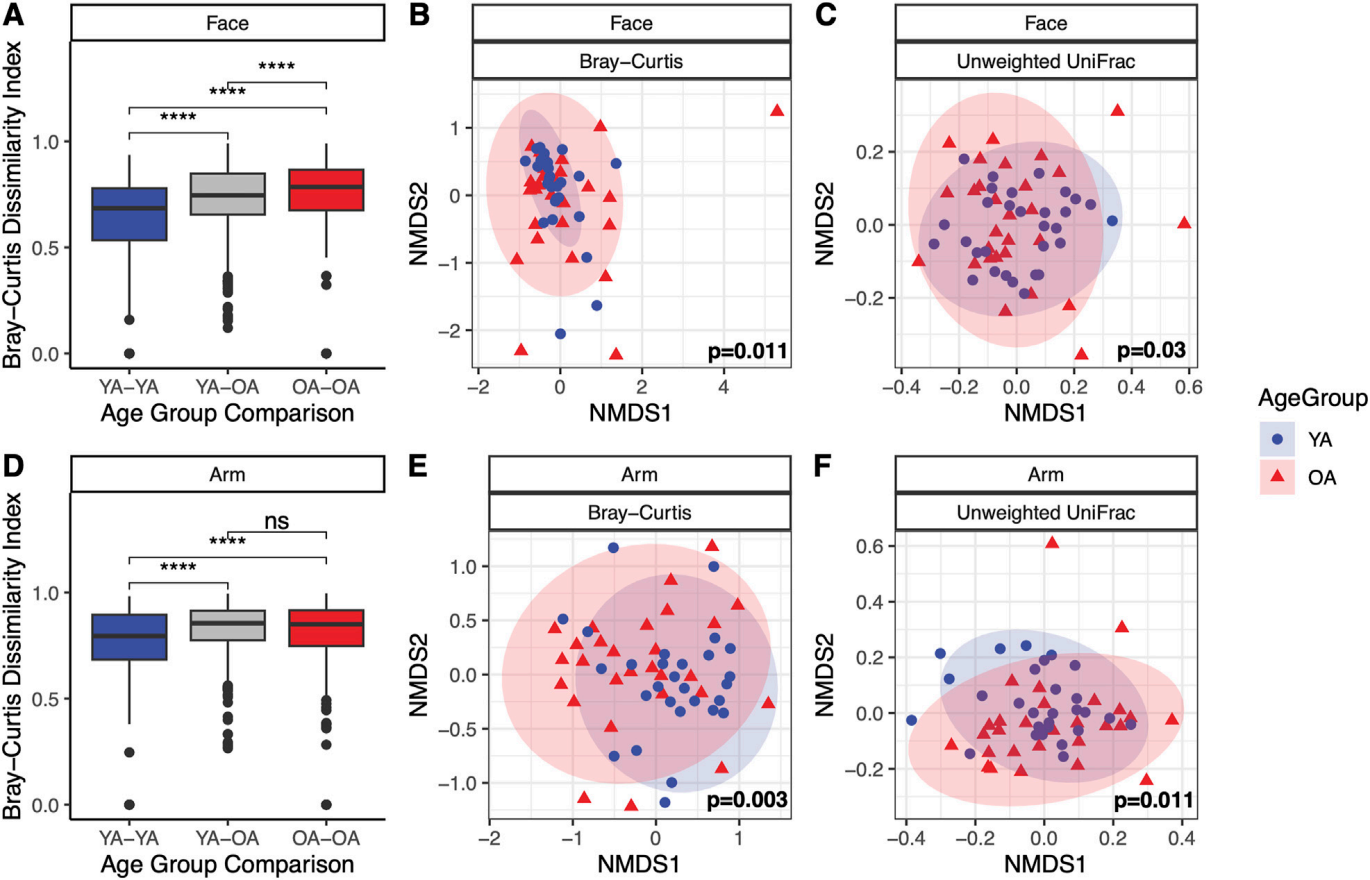
Significant differences in microbial community composition with age in the face and arm microbiome. Microbial community composition shows significant variation between young age (YA) and old age (OA) groups in both the face and arm. **(A,D)** Bray-Curtis dissimilarities between individuals within the same and different age groups for the face and arm, respectively. Statistical significance was calculated using the Wilcoxon rank sum test (**** *p*-value <0.0001, ns = not significant). **(B,E)** NMDS ordination of the Bray-Curtis dissimilarity matrix for the face and arm, respectively. **(C,F)** NMDS ordination of the unweighted UniFrac distance matrix for the face and arm, respectively. Statistical significance for compositional differences was calculated using PERMANOVA, with the *p*-value indicated within each ordination plot. Ellipses represent a 95% confidence interval for each age group. Blue circles represent the young age (YA) group and red triangles represent the old age (OA) group.

In assessing beta diversity, microbial community composition differed significantly between age groups for both the face and the arm using Bray-Curtis dissimilarity and unweighted UniFrac (p < 0.05), with notably higher interpersonal variation in the old age group compared to the young age group (Bray-Curtis, p < 0.001) (Figure 2). Weighted UniFrac exhibited significant differences in community composition for the face but not the arm between age groups (Supplementary Figures S2A, B). These findings suggest that differences in microbial communities between age groups for the face may be driven by variations in species abundances and evolutionary relationships among abundant taxa, as well as the presence/absence of low-abundance, phylogenetically distinct taxa. In contrast, for the arm, the compositional differences may be primarily due to shifts in the abundances of closely related taxa and the presence/absence of specific, phylogenetically unique taxa. When examining community composition across all samples, significant differences in Bray-Curtis dissimilarity were observed between the face and arm (p = 0.001, R^2^ = 0.0679) as well as between young age and old age (p = 0.001, R^2^ = 0.0287), with skin site having a larger impact on community composition differences than age group (Supplementary Figure S2C). Furthermore, evaluation of microbial community composition by gender revealed significant differences in Bray-Curtis dissimilarity for female participants across both the face and arm, however no significant differences were observed for male participants (Supplementary Figures S1D–G), suggesting that gender plays an important role in driving skin microbial community differences with age.

### Age-related taxonomic shifts across skin sites

**FIGURE 3:**
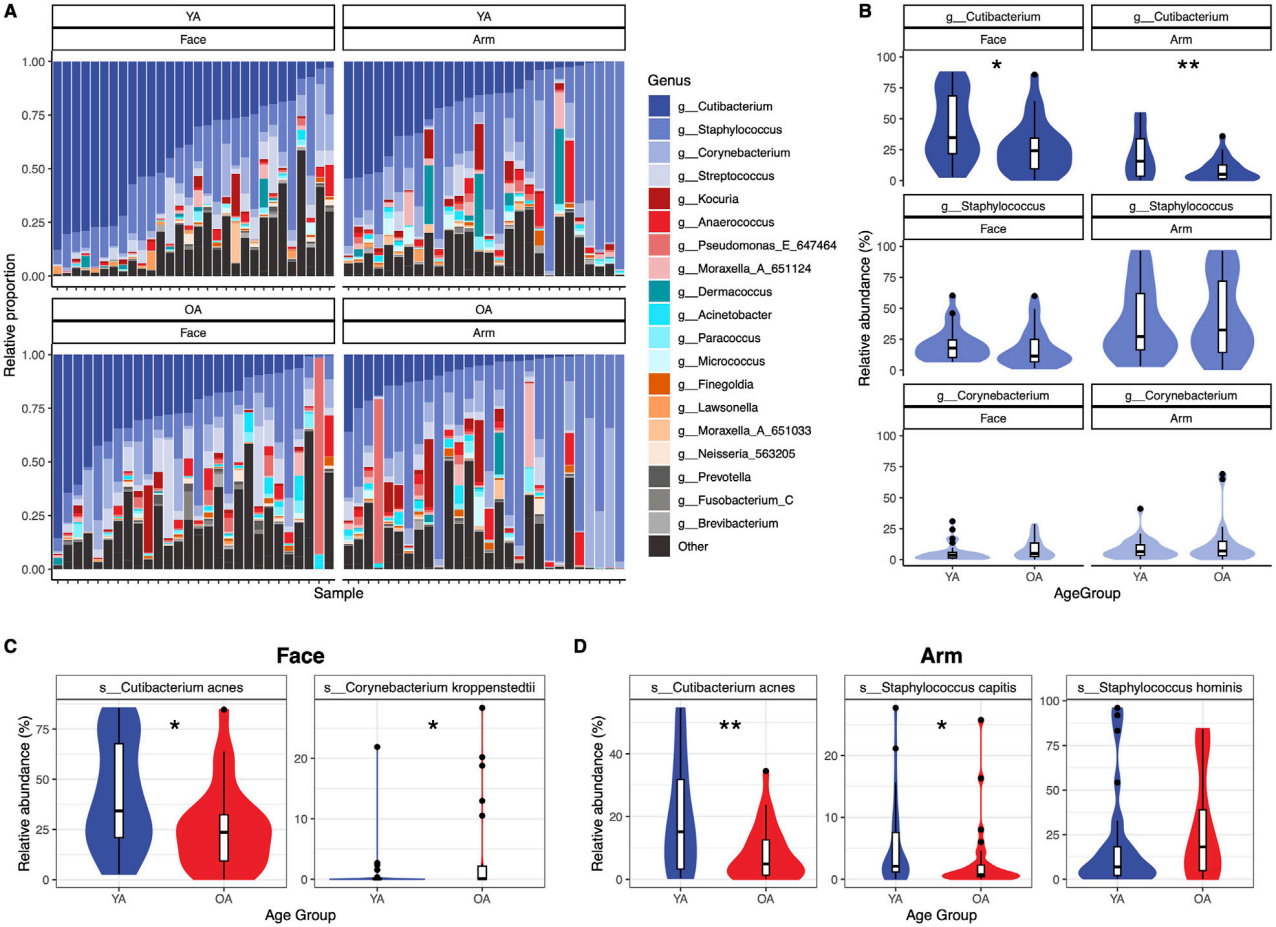
Genus- and species-level variation of abundant skin taxa between age groups. Significant differences are observed between age groups for abundant genera and species on the skin. **(A)** Relative proportions of the top twenty genera are presented as stacked bar plots for each sample within each age group and skin site, with samples ordered by *Cutibacterium* abundance from highest to lowest. Genera not included within the top twenty genera are grouped into “Other”. **(B)** Relative abundance differences between *Cutibacterium, Staphylococcus*, and *Corynebacterium* within each age group and skin site. Genera are colored according to the legend in **(A)**. **(C)** Relative abundance differences for species of interest for the **(C)** face and **(D)** arm. Statistical significance was calculated using the Wilcoxon rank sum test * *p*-value <0.05, ** *p*-value <0.01. YA = young age group, OA = old age group.

At the phylum level, the face demonstrated a significant decrease with age in Actinobacteriota (Actinobacteria) alongside a significant increase in Pseodomonadota (Proteobacteria) and taxa outside of the Actinobacteriota, Pseudomonadota, and Bacillota (Firmicutes) phyla (Supplementary Figure S3). The arm did not exhibit significant age-related phylum-level differences. At the genus level, microbial community composition exhibited high interpersonal variation (Figure 3A). Among the top three most abundant genera on the skin, *Cutibacterium*, *Staphylococcus*, and *Corynebacterium*, both skin sites showed a significant decrease in *Cutibacterium* in the old age group (Face *p* = 0.033, Arm *p* = 0.007), with minimal differences in *Staphylococcus* and *Corynebacterium* abundances (Figure 3B). Interestingly, this pattern was gender-dependent; only female participants exhibited a significant decrease in *C. acnes* with age for both skin sites, in addition to a significant decrease in *Staphylococcus* on the face (Supplementary Figure S4).

Differential abundance testing identified 4 ASVs that were differentially abundant between age groups on the face: *C. kroppenstedtii, Phocaicola vulgatus, Streptococcus* sp., and *Streptococcus thermophilus* (*q* < 0.05) (Supplementary Figure S5A)*.* On the arm, 19 ASVs were differentially abundant: *Corynebacterium amycolatum, Corynebacterium dentalis,* five *Cutibacterium acnes* ASVs, one *Lawsonella* ASV, *Moraxella cinereus*, three *Staphylococcus capitis* ASVs, four *Staphylococcus hominis* ASVs, two *Staphylococcus* ASVs, and a *Weeksellaceae* ASV (*q* < 0.05) (Supplementary Figure S5B).

All ASVs assigned to *C. acnes, C. kroppenstedtii, S. hominis,* and *S. capitis,* which were selected based on their differential abundance results as well as relevance to skin health, were aggregated to the species level for further analysis. *C. acnes* has been previously reported to decrease with age, attributed to a decrease in sebum present on the skin (Larson et al., 2022; Garlet et al., 2024; Zhou et al., 2023). We observed that *C. acnes* is significantly reduced both on the face and the arm (−16.1% and −11.4% relative abundance, respectively) in the old age group compared to the young age group (Face *p* = 0.024, Arm *p* = 0.0078) (Figures 3C,D). Similarly, *C. kroppenstedtii* has also been previously found to increase on the skin with age and has been reported to be associated with rosacea (Garlet et al., 2024; Dimitriu et al., 2019; Zhou et al., 2023; Rainer et al., 2020). On the face, *C. kroppenstedtii* was significantly increased in the old age compared to the young age group (*p* = 0.022, +3.51% mean relative abundance) (Figure 3C). Lastly, we examined levels of *S. capitis* and *S. hominis*, as several ASVs assigned to these species were differentially abundant between age groups on the arm (Supplementary Figure S5B)*. S. capitis* showed a significant decrease in old age compared to young age (*p* = 0.026, −2.12% mean relative abundance), while *S. hominis* trended towards an increase in old age (*p* = 0.15, +9.03% mean relative abundance) (Figure 3D). Assessing differences by gender revealed that the significant decrease with age observed for *C. acnes* on the face and arm and *S. capitis* on the arm is largely driven by the female participants (Supplementary Figures S4B, C). However, *S. hominis* shows an increase in old age for both genders, suggesting a gender-independent mechanism. Overall, numerous taxa demonstrate age-specific changes, which notably are distinct between skin sites.

### Loss of microbial community network structure in old age

To evaluate the impact of age and skin site on microbial interactions and the overall microbial community network, we employed the statistical method SPIEC-EASI to infer microbial associations between skin microbiome members of the different age groups and skin sites. We selected features found in at least 30% of all face or arm samples, with a feature in this instance referring to ASVs agglomerated to the species level or retained at their original taxonomic level if a species-level assignment was unavailable. This resulted in 194 features for the face and 144 features for the arm being included in the analysis.

**FIGURE 4:**
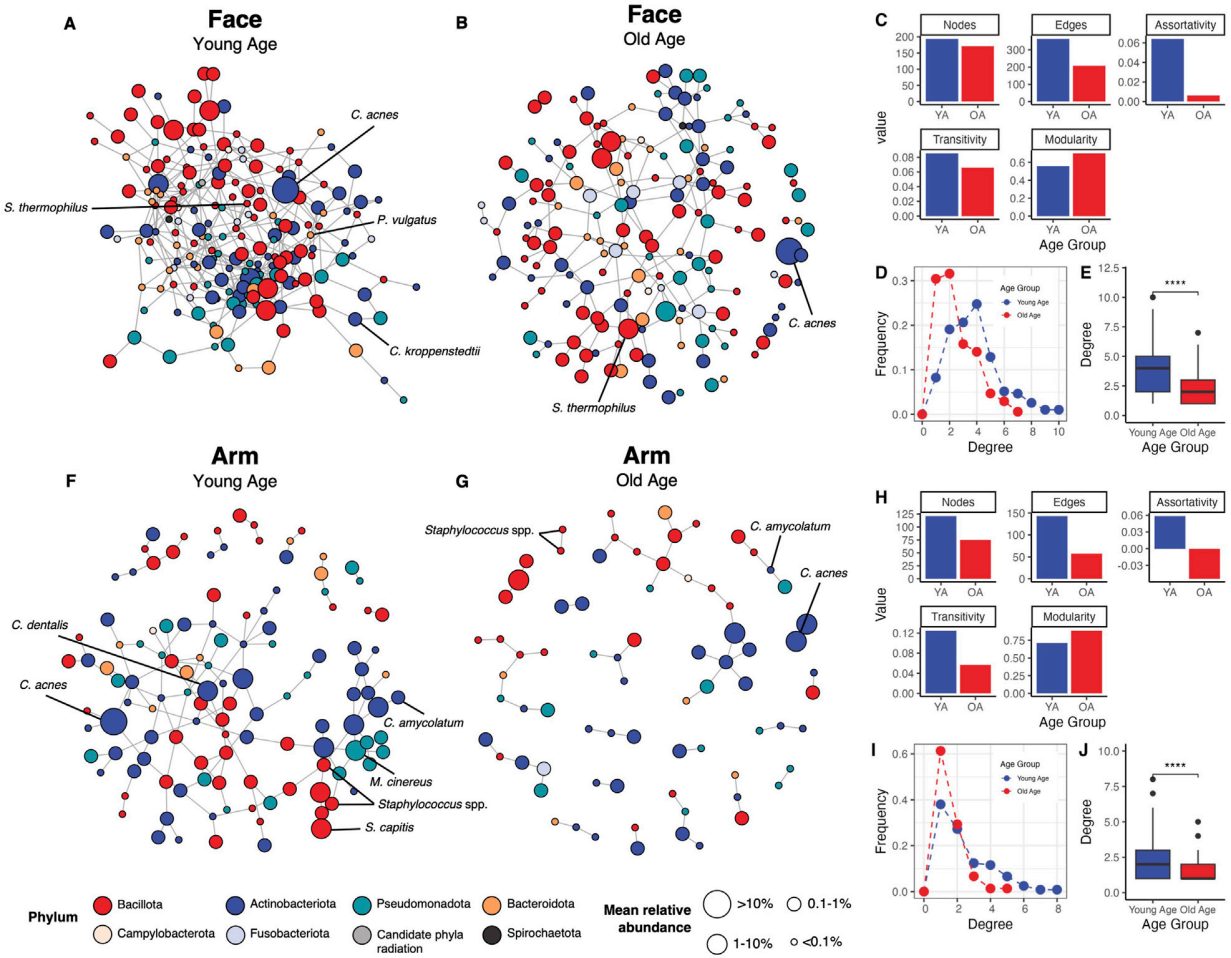
Microbial association networks show increased sparsity and reduced connectivity with age. The SPIEC-EASI statistical method was used to identify microbial associations between skin microbiome members for each age group and skin site. Networks for the face in the **(A)** young age group and **(B)** old age group are shown, alongside **(C)** network statistics (number of nodes, number of edges, degree assortativity, transitivity, and modularity), **(D)** degree frequency distributions, and **(E)** degrees per node comparison for the face. Similarly, networks for the arm in the **(F)** young age group and **(G)** old age group are presented, with corresponding **(H)** network statistics, **(I)** degree frequency distributions, and **(J)** degrees per node comparison for the arm. Nodes represent microbial features (ASVs agglomerated to the species level or ASVs retained at their original taxonomy level if a species-level assignment was unavailable), which are colored by their phylum assignment and sized by their mean relative abundance within the skin site and age group collectively. Edges represent associations identified between features. Only features with at least one association are included in the networks. Nodes representing taxa of interest or differentially abundant taxa are indicated in black text. Statistical significance was calculated using the Wilcoxon rank sum test (**** *p*-value <0.0001). YA = young age group, OA = old age group.

For both skin sites, the microbial networks in old age had fewer nodes and edges compared to the young age networks, indicating fewer associating taxa within the network with age (Figures 4A–C,F–H). In addition, the old age networks showed reduced degree assortativity and transitivity [a measure of the preference for a node to attach to other nodes with similar degree count and the overall probability for the network to have adjacent nodes interconnected, respectively (Röttjers and Faust, 2018)], increased modularity [a measure of the strength of division of a network into modules (Röttjers and Faust, 2018)], and a lower frequency of high-degree nodes compared to the young age group (Figures 4C,D,H–I). Both old age networks had significantly reduced node degrees compared to the young age networks (*p* < 0.001) (Figures 4E,J). The face network in young age showed particularly high connectivity, with all 194 features (nodes) having at least one association with other features. In contrast, the old age network for the face exhibited a less dense structure, with only 171 associating features. Similarly, the arm network in old age showed a marked shift from the young-age network, presenting a highly sparse network composed of numerous modules with limited connectivity (121 vs. 75 associating features in young versus old age, respectively). This reduction in connectivity could indicate a microbial community in old age that exhibits reduced interaction and cooperation among microbial members. Interestingly, differentially-abundant and skin health-related taxa that showed significant differences with age (Figure 3; Supplementary Figure S5) also were more prevalent and connected within the network in young age compared to old age for both skin sites. For example, *C. acnes* associated with numerous other taxa within the young age networks, but only formed a single association in the old age networks. Overall, these findings suggest that in old age, the microbial communities of the face and arm become less robust, less stable, and more fragile.

### DMM clustering reveals age-related microbiome profiles

**FIGURE 5:**
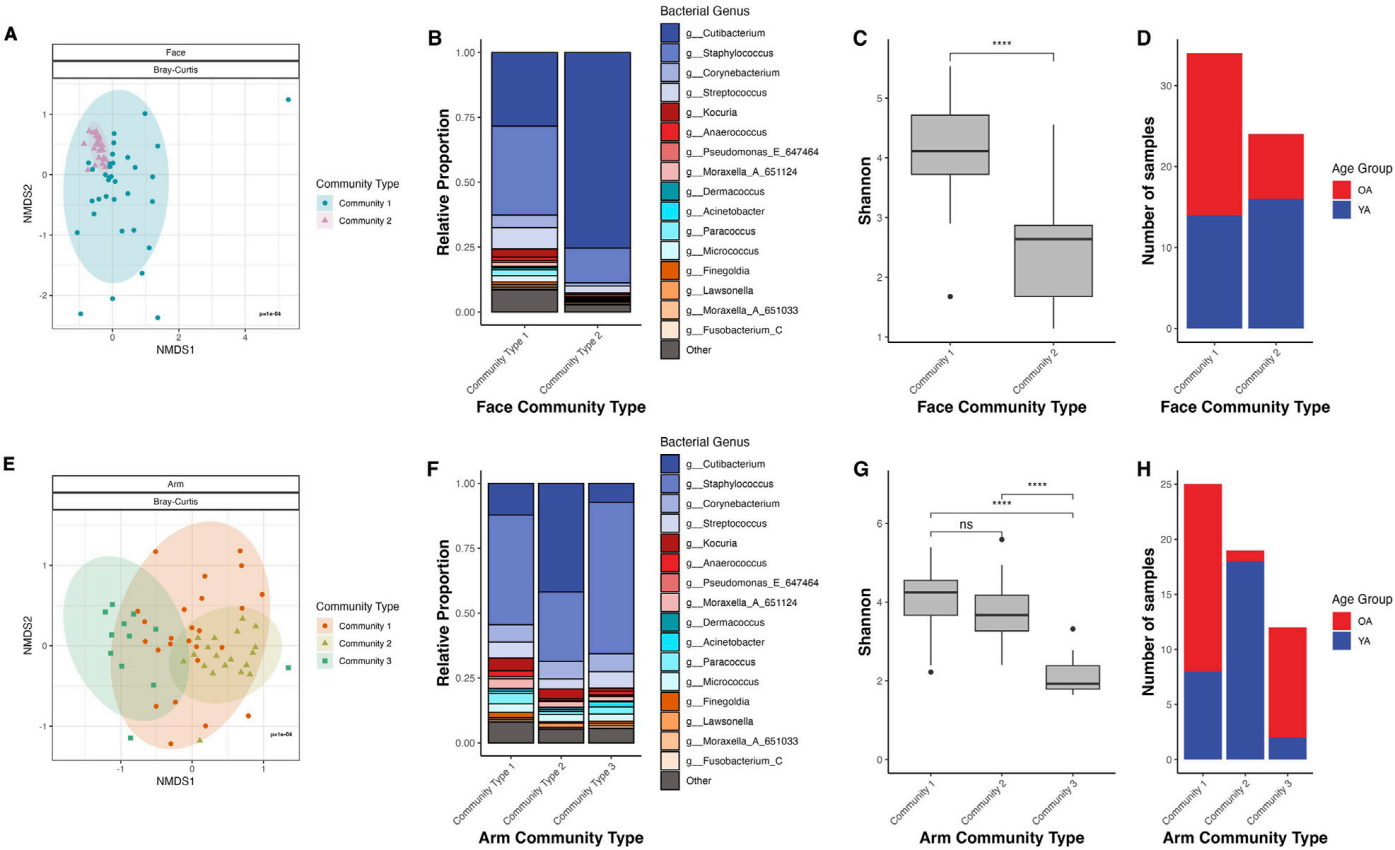
Dirichlet multinomial mixtures (DMM) clustering identifies age-specific microbiome profiles for the face and arm. **(A,E)** NMDS ordination of the Bray-Curtis dissimilarity matrix for the face and arm, respectively. Points are colored and shaped by their DMM community type. Ellipses represent a 95% confidence interval for each age group. **(B,F)** Stacked bar plot representing the genus-level microbiome community composition of each community type as determined through Dirichlet multinomial mixtures (DMM) in the face and arm samples, respectively. Taxa not within the top twenty most abundant genera are grouped into “Other”. **(C,G)** Shannon diversity for the community types in face and arm samples, respectively. Statistical significance was calculated using the Wilcoxon rank sum test (**** *p*-value <0.0001). **(D,H)** Number of samples that are assigned to each community type, colored by age group, in the face and arm samples, respectively. YA (blue) = young age, OA (red) = old age.

To explore the relationship between microbial community composition and age, we performed mathematical modeling of ASV frequencies using Dirichlet multinomial mixtures (DMM) to uncover potential age-related microbiome structures across participants. DMM clustering separated the face samples into two community types with significantly different microbial community compositions (Bray-Curtis dissimilarity, PERMANOVA *p* = 1E-04) (Figure 5A). Community Type 1 exhibited high Shannon diversity, with *C. acnes* and *Staphylococcus epidermidis* being the primary drivers of this community type (Figures 5B,C; Supplementary Figure S6A). In contrast, Community Type 2 was significantly less diverse than Community Type 1 (*p* < 0.001) and was dominated by *C. acnes* (Supplementary Figure S6B). Community Type 2 had a predominance of young age group individuals (66.7%) compared to 33.3% from the old age group, whereas Community Type 1 was more balanced with 41.2% from the young age group and 58.8% from the old age group (Figure 5D). When comparing facial community type distribution by gender for both Community Type 1 and Community Type 2, female participants mirrored the overall cohort’s age group distribution, while the male participants had a relatively equal age group distribution (Supplementary Figure S7A), suggesting that gender is an important driving factor in face microbiome shifts with age.

For the arm, DMM identified three distinct community types, which exhibited significant differences in microbial community composition (Bray-Curtis dissimilarity, PERMANOVA *p* = 1E-04) (Figure 5E). Community Type 1 exhibited high Shannon diversity and was predominantly driven by *C. acnes*, *S. hominis*, and *S. epidermidis,* appearing in 68% of samples from the old age group (Figures 5F–H; Supplementary Figure S6C). Community Type 2 demonstrated similar diversity to Community Type 1, however was largely driven by *C. acnes* alone (Supplementary Figure S6D). Notably, all but one sample assigned to Community Type 2 was from the young age group. Lastly, Community Type 3, dominated by *S. homini*s, showed significantly reduced Shannon diversity compared to the other community types (*p* < 0.001), with 83.3% of samples from the old age group (Supplementary Figure S6E). When comparing by gender, the age distribution patterns for both male and female participants mirrored the overall distribution, with Community Types 1 and 3 being old age-dominant and Community Type 2 being young-age dominant (Supplementary Figure S7B). This suggests that for the arm, age-related microbial community shifts are gender-independent.

### The face microbiome exhibits a shift in predicted functional potential with age

**FIGURE 6:**
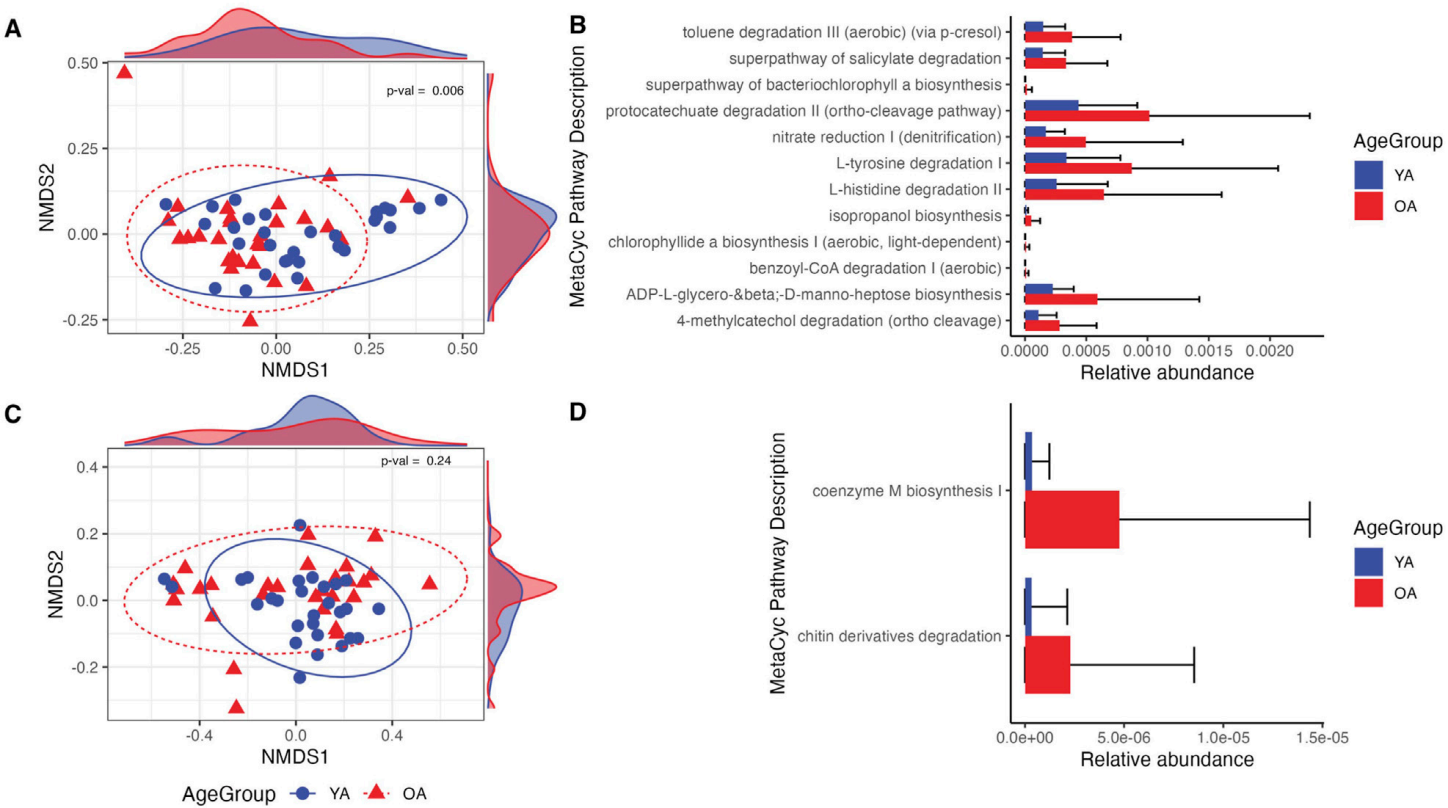
Differences in predicted microbial functional potential are more prominent in the face than the arm in old age. **(A,C)** NMDS ordination of the Bray-Curtis dissimilarity matrix computed on MetaCyc pathway relative abundances for the face and arm samples, respectively. Density plots within the plot margins represent the density of points along the x and y-axes for each age group. **(B,D)** Relative abundances of MetaCyc pathways determined to be differentially abundant between young age (YA) and old age (OA) groups for the face and arm, respectively. All pathways shown are statistically significant with *q*-value <0.05. YA (blue) = young age, OA (red) = old age. Statistical significance for pathway compositional differences was calculated using PERMANOVA, with the *p*-value indicated within each ordination plot.

PICRUSt2 was utilized to assess the functional potential of the face and arm samples across age groups, predicting MetaCyc metabolic pathways based on ASV-level data. For the face, there was a significant shift in the predicted functional profile between young age and old age (*p* = 0.006), and numerous metabolic pathways were found to be more abundant in old age compared to young age (*q* < 0.05) (Figure 6A). These include functions related to aromatic hydrocarbon and pollutant degradation (aerobic toluene degradation III via p-cresol, superpathway of salicylate degradation, protocatechuate degradation II, aerobic benzoyl-CoA degradation I, and 4-methylcatechol degradation), amino acid degradation (L-tyrosine and L-histidine), and lipopolysaccharide biosynthesis (ADP-L-glycero-β-D-manno-heptose biosynthesis) (Figure 6B). In contrast, there was not an observed shift in predicted metabolic function of the microbial communities between age groups for the arm (Figure 6C). Two pathways, coenzyme M biosynthesis and chitin derivatives degradation, were significantly more abundant in old age compared to young age (*q* < 0.05), although the relative abundances of these pathways were very low (Figure 6D).

## Discussion

The aging process directly impacts skin appearance and function, resulting in distinct changes that range from visible signs of aging, such as fine lines and wrinkles, to increased susceptibility to skin conditions and infections (Tobin, 2017). These physiological changes are accompanied by shifts in the microbial populations that inhabit our skin, necessitating a better understanding of the consequences that these altered microbial communities have on skin health, particularly across different body sites. In our study, we find that the skin microbiome of the face and arm undergo significant yet distinct shifts with age, highlighting the site-specific variation in microbiome dynamics that are impacted by the aging process.

The face is a highly exposed skin region that is chronically exposed to external factors such as UV radiation and air pollution, both of which significantly contribute to signs of skin aging (Krutmann et al., 2017). We observed strong shifts in the microbiome of the face between young and old age groups, including increased alpha diversity, significant microbiome community composition shifts characterized by a large reduction in *C. acnes*, altered network topology, and predicted functional changes. The arm, while being a less exposed skin site than the face (Bulliard et al., 2007), also exhibited significant changes in the microbiome between age groups, albeit less pronounced. These included shifts in microbiome community composition characterized by a decrease in *C. acnes* and shifted *Staphylococcus* populations, as well as an altered network structure. Thus, skin sites with differential exposure to environmental aggressors show distinct skin microbiome shifts, suggesting that these differences could be driven in part by differences in intrinsic versus extrinsic skin aging factors. Indeed, differential exposure to polycyclic aromatic hydrocarbons (PAH), a class of organic pollutants in ambient air, has been shown to impact the skin microbiome, metabolome, and clinical skin parameters, with high exposure to PAH associated with biodegrading skin bacteria, dry skin, and hyperpigmentation (Leung et al., 2023; Leung et al., 2020; Misra et al., 2021). Similarly, exposure to UVA and UVB radiation, which have profound impacts on skin aging (Krutmann et al., 2017), has been observed to immediately alter microbiome composition (Burns et al., 2019). Therefore, the interplay between the skin, its microbiome, and aging are likely to be multi-faceted and dynamic, influenced by a combination of genetic factors, environmental exposures, and lifestyle choices.

A notable reduction in *C. acnes* was observed in old age for both skin sites (Figures 3C,D), supporting previous studies that similarly found this species to be a biomarker negatively associated with aging (Larson et al., 2022; Garlet et al., 2024; Zhou et al., 2023). Reduced sebum availability likely leads to a decrease in *C. acnes*, which plays a key role in maintaining skin pH through production of free fatty acids from sebum metabolism, contributing to the skin’s acidic nature and its protective resistance against unwanted microorganisms (Swaney and Kalan, 2021). While often associated as an etiological contributor to acne, *C. acnes* is a ubiquitous skin commensal that has key roles in immunomodulation, epithelial barrier regulation, lipid synthesis, and protection against pathogens (Almoughrabie et al., 2023; Claesen et al., 2020; Nagy et al., 2006; Agak et al., 2018; Nakamura et al., 2020; Sanford et al., 2016). As a result, depletion of *C. acnes* is likely to lead to hyperdiversification as a result of increased niche availability and reduced colonization resistance. This can provide rationale for the observed increase in interpersonal variation seen on the face with old age; diminished colonization resistance could allow for an increase in presence and abundance of non-specific taxa, particularly those that are transient and environmental, leading to high interpersonal variability of the microbiome. Indeed, predictive functional analysis of the face microbiome revealed increased abundances of functions involved in aromatic hydrocarbon/pollutant degradation (Figure 6), functions that are distributed among environmental microorganisms due to the widespread presence of these compounds in nature (Fuchs et al., 2011; Ke et al., 2023; Porter and Young, 2014; Parales et al., 2008). Increased abundance of bacteria with biodegradation capabilities has also been associated with high-level PAH exposure (Leung et al., 2023), providing a possible link in the present study between pollution exposure, the skin microbiome, and aging.

Our study also revealed gender-specific differences in how the microbiome differs with aging across different body sites. For the face, the microbiome changes observed in female participants largely mirrored the trends seen in the overall cohort results, characterized by a significant shift in community composition and diversity with age (Supplementary Figure S1). This may be attributed to the hormonal shifts experienced by women during menopause, which lead to a notable reduction in sebum production (Zouboulis and Boschnakow, 2001). Given that sebum availability influences growth of the skin microbiota (Swaney et al., 2023), these physiological changes are likely important drivers of the pronounced differences observed in the female microbiome with age. Conversely, the typically higher and more stable sebum production in males (Townsend and Kalan, 2023; Pochi et al., 1979) may provide a more consistent and robust microbial niche on the face. This could buffer against the age-related microbial changes seen in females, potentially explaining the more limited age-related differences observed in the male facial microbiome. However, on the arm, the aging-related microbial changes were similar across genders, suggesting that the less sebaceous environment of the arm is less influenced by gender-specific physiological factors. These findings underscore the importance of considering both skin site and gender in future studies on the skin microbiome and aging.

Our clustering analysis revealed the existence of age-driven microbiome profiles for both the face and arm. In particular, two facial microbiome types were identified using DMM, with high diversity/low *C. acnes* more frequent in old age and low diversity/high *C. acnes* more prevalent in young age (Figures 5A–D). These findings support previous efforts to assign facial skin microbiomes to cutotypes by applying Partitioning Around Medoids (PAM) clustering, which similarly identified a *Cutibacterium-*cutotype that exhibited low Shannon diversity and whose proportion declined with age across individuals (Sun et al., 2024). Therefore, *C. acnes* may play a significant role in maintaining a core, balanced microbial community in younger individuals, with its decline contributing to increased diversification and susceptibility to perturbation and dysbiosis with age (Larson et al., 2022). Interestingly, we observed two distinct old age-associated microbiome types for the arm; their compositional profiles were both characterized by a predominance of *S. hominis*, but differed in respect to their overall community diversity (Figures 5E–H). This divergence suggests that aging may not only shift the dominant taxa, but may also influence ecological stability and resilience of these communities. Indeed, microbial association analysis revealed a stark shift in structure of the microbial networks between young and old age for both skin sites (Figure 4), highlighting a less robust and more fragile microbiome with age. This has been observed in previous studies, which have similarly observed diminished microbial community stability and resilience in older populations (Kim et al., 2022; Kim et al., 2019). Overall, these findings suggest that aging not only disrupts the core, *C. acnes*-dominant microbiome, but also promotes a less predictable and more perturbation-susceptible microbial community that notably is skin-site specific.

Our findings highlight the potential role of *S. capitis* and *S. hominis* as key differentiating species between young and old age on the arm, with *S. capitis* more abundant in young age and *S. hominis* more abundant in old age (Figure 3D; Supplementary Figure S6). *S. capitis*, similar to *C. acnes*, thrives in lipid-rich skin regions (Chong et al., 2022), which may explain the observed decrease in abundance of this species with age. *S. hominis* is the second most frequently isolated coagulase-negative staphylococci from healthy human skin and is known for its ability to produce antimicrobial peptides as well as thioalcohol from apocrine gland secretions, contributing to body odor (Severn et al., 2022; Rudden et al., 2020). Interestingly, old-age individuals have a characteristic body odor (Mitro et al., 2012), suggesting a link between elevated levels of this bacterial species in old age and dermal body odor. While the effect of a *S. capitis*-to-*S. hominis* shift on skin health is unknown, it suggests that *S. hominis* may opportunistically colonize the skin in response to decreased competition and altered ecological niches resulting from aging, potentially exerting influence on microbial homeostasis and the skin’s local immune response. In support of this notion, *S. hominis* was previously found to be inversely correlated with sebum content and positively correlated with staphylococcal alpha diversity (Ahle et al., 2022), highlighting the potential importance of skin physiology and microbial community context in *S. hominis* expansion. *S. hominis* has also shown to have inhibitory properties against *C. acnes* (Ahle et al., 2022), suggesting interspecies competition may also play a role. Our study was limited to species-level interrogation of these bacteria, however, high strain heterogeneity and intra-species diversity has been well-documented for *S. capitis*, *S. hominis*, and *C. acnes* (Joglekar et al., 2023; Conwill et al., 2022), warranting future investigation into the role of strain diversity in the microbiome and aging.

Aging has been shown to impact not only the skin microbiome, but also the microbiome of other body sites such as the oral cavity and gastrointestinal tract (Larson et al., 2022; Huang et al., 2020; Sarafidou et al., 2024; Ghosh et al., 2022). As a part of this study, we also concurrently collected saliva samples for microbiome analysis from the same cohort (manuscript in progress). Characterizing the microbiota of these two distinct body sites will allow for even more in-depth insight into the impact of aging on the human microbiome, notably within the same population. Furthermore, this will provide the unique opportunity to integrate microbial data across body sites to investigate relationships between the skin and oral microbiota, particularly given the observation that oral bacteria have previously been associated with skin microbiome diversification in aging (Shibagaki et al., 2017).

While our study provided a detailed overview of how aging may modulate the bacterial members of the skin microbiome, it was limited by the use of 16S rRNA gene sequencing, which excludes non-bacterial microorganisms and does not provide strain-level resolution. Future efforts should employ metagenomic sequencing and additional techniques such as metatranscriptomics and metabolomics to gain a greater depth of understanding into the mechanisms of microbial function and strain specificity in skin aging. Furthermore, our study did not measure parameters of skin health such as moisture levels, elasticity, and the presence of visible signs of aging. Inclusion of these metrics may provide more defined relationships between microbiome shifts and aging. For example, relationships have been identified between the skin microbiota and clinical signs of aging such as grade of Crow’s feet wrinkles (Myers et al., 2023; Garlet et al., 2024) and dark spot area (Shibagaki et al., 2017; Li et al., 2020), as well as skin characteristics including sebum content, sebaceous gland area, transepidermal water loss (TEWL), natural moisturizing factor (NMF), antimicrobial peptides (AMPs), and lipids (Howard et al., 2022; Kim et al., 2022; Sun et al., 2024; Kim et al., 2019). An additional limitation of the study is the potential influence of confounding variables that we did not control for, which include lifestyle factors (e.g., cosmetic use, timing of last wash) and health-related factors (e.g., underlying health conditions, medication). Lastly, our study was limited by collection of the microbiome at a single time point. Future efforts should employ longitudinal studies to better understand temporal dynamics of the microbiome during the aging process, which may help to elucidate how changes in the skin microbial communities correlate with the progression of skin aging.

In summary, these findings provide comprehensive insights into the microbiome of aging skin in a UK-based population, highlighting the shift from a core, stable community driven by *C. acnes* in young individuals, to a more variable and less robust community in older individuals. This work also supports and complements previous studies of the aging skin microbiome carried out on North America- and Asia-based populations, highlighting the connection between the skin microbiota and the physiology of skin aging across ethnicities and geography. By comparing the microbiome of distinct skin sites, our study also underscores the importance of considering skin site specificity when studying the impact of aging on skin microbiome dynamics, with both intrinsic and extrinsic factors of aging likely playing significant roles. We also demonstrate the potential of microbiome typing techniques to unravel complex microbial interactions and microbiome profiles associated with age, emphasizing the opportunity for advanced techniques such as machine learning in generating more personalized and precision interventions tailored to the needs of an individual’s aging skin. Expanding these advanced methods to include other omics data, skin health parameters, and longitudinal studies has the potential to further our understanding of the intricate interplay between the skin microbiota, skin health, and the aging process. Ultimately, our findings highlight a significant opportunity for developing innovative and targeted next-generation anti-aging solutions, such as prebiotic or postbiotics treatments, to restore a more youthful, stable skin microbiome and thereby enhance overall skin health.

## Methods

### Study design

Ethical approval for the study was granted by Aston University’s College of Health and Life Sciences ethics committee (reference number HLS21008). Participants were recruited via advertisement through the Aston Research Centre for Health in Ageing (ARCHA), Aston University, and online social media channels. All participants gave informed written consent prior to sample collection. Initial participant screening was performed using a survey hosted on Qualtrics, during which basic demographic information was collected including age, gender, ethnicity, and postcode. Participants were required to speak and understand written English and were required to be aged between 20 and 40 or 60–80 years old. Participants who did not meet these criteria or who were pregnant were excluded. The specified age groups were selected to create distinct contrast between young and old populations and minimize the potential confounding effects of variables that might be present in a more heterogeneous middle-aged cohort, such as significant lifestyle changes and hormonal shifts (e.g., perimenopause). Additional metadata was collected at the time of sampling that included health, lifestyle, and geographical information, however this information was not used for study inclusion or exclusion. In total, 59 participants from Birmingham, United Kingdom were included in the study. Thirty participants were included in the young age (YA) group, and 29 participants were included in the old age (OA) group.

### Sample collection

Sample collection and processing was performed at Aston University (Birmingham, United Kingdom). Participants continued their normal daily health and hygiene routine and did not follow any washout guidelines before sampling, reflecting a non-controlled, more representative microbiome state. A sampling solution was made using phosphate buffered saline modified with Tween^®^ 20 to a final concentration of 0.1% (v/v). Sampling templates with a 5 cm × 5 cm area were made from SILASTIC™ RTV-4136-M Liquid Silicone Rubber (Dow, Midland, Michigan). The sampling solution and templates were sterilized by autoclaving at 121 °C for 15 min prior to use.

Microbiome samples were collected from the right cheek and from the right antecubital fossa (interior of the inner elbow). For each area a sterile viscose swab (Scientific Laboratory Supplies, Nottingham, United Kingdom, catalog number: SWA3112) was soaked in 2 mL of sampling solution and used to swab the participants skin within the sample area in a constant “zigzag” pattern. The swab head was then stored inside the 2 mL sampling solution for storage at 2–8 °C prior to processing. Blank control swabs were collected by soaking the swab in sampling solution and processed in a manner identical to the skin swabs.

### Preservation and processing of samples

Initial processing of samples occurred within 24 h of sampling. DNA extraction was performed on the samples using Qiagen DNeasy PowerSoil Pro Kit (QIAGEN, Hilden, Germany, catalog number: 47014) following the manufacturer’s protocol. DNA concentration was measured using a NanoDrop 1000 spectrophotometer (Thermo Fisher, United Kingdom), following storage of the DNA extracts at −80 °C. For transit prior to sequencing the DNA extracts were dried using an Eppendorf Concentrator plus at 30 °C for 45 min.

### 16S rRNA gene sequencing

Sequencing of the V1-V3 hypervariable region of the 16S rRNA gene was carried out by the Rutgers University Genomic Center. The V1-V3 region was amplified using NEB HiFidelity DNA polymerase (New England Biolabs) with the 28F (5′ GAGTTTGATCNTGGCTCAG) and 519R primers (5′ GTNTTACNGCGGCKGCTG). Barcoding was performed using Nextera XT dual index primers (Illumina) and NEB HiFidelity DNA polymerase (New England Biolabs). Libraries were sequenced on an Illumina MiSeq 2 x 300 flow cell.

### Microbiome data analysis

#### Sequence processing

Raw FASTQ sequencing data was imported into QIIME2 (version 2024.2.0) (Bolyen et al., 2019). Quality assessment identified primers and low-quality regions within the forward and reverse reads, therefore quality trimming was applied as follows: forward reads were trimmed 19 bp and truncated at 291 bp, and reverse reads were trimmed 18 bp and truncated at 261 bp, providing 22 bp of read overlap between forward and reverse reads. The DADA2 plugin within the QIIME2 environment was used to generate an ASV feature table with 23,004 ASVs (Callahan et al., 2016). Four samples with read counts below 10,000 were removed following quality processing, resulting in 114 total microbiome samples with an initial median read count of 114,226 per sample for downstream filtering.

#### Taxonomic classification

To achieve improved species-level resolution with taxonomic assignment, a custom Naive Bayes classifier was trained on the extracted V1-V3 region of the Greengenes2 16S database (McDonald et al., 2024). Briefly, the V1-V3 region was extracted from the preformatted Greengenes2 backbone sequences (2022.10.backbone.full-length.fna.qza) using the 28F forward (GAGTTTGATCNTGGCTCAG) and 519R reverse (GTNTTACNGCGGCKGCTG) primers, with a 400 bp minimum length and 550 bp maximum length required for the extracted sequences (*qiime feature-classifier extract-reads*) (McDonald et al., 2024). Extracted sequences were then dereplicated using the “uniq” method from the RESCRIPT QIIME2 plugin (*qiime rescript dereplicate*). A Naive Bayes classifier was trained on this set of dereplicated V1-V3 sequences (*qiime rescript evaluate-fit-classifier*) (Robeson et al., 2021), followed by taxonomic assignment of ASVs using the classifier (*qiime feature-classifier classify-sklearn*).

#### Decontamination

To ensure a high-quality, robust dataset, decontamination of the samples was performed. Because the Greengenes2 2022.10 backbone taxonomy does not explicitly represent mitochondria and chloroplast, a prefiltering step was required to eliminate these contaminant ASVs. Here, ASVs were classified using a V1-V3 Naive Bayes classifier trained on the Silva 138 SSURef NR99 full-length database (Quast et al., 2013). This step identified 190 ASVs classified as chloroplast or mitochondria, which were then removed from the Greengenes2-classified ASV table. Subsequently, manual filtering was applied to remove ASVs unassigned at the domain level, ASVs assigned as Archaea or Eukaryota, and ASVs assigned as *Pelomonas* or *Bradyrhizobium*–common contaminants, especially in low biomass samples (Salter et al., 2014; Eisenhofer et al., 2019) – which collectively accounted for 3,964 ASVs removed. A final decontamination step using the combined frequency- and prevalence-based approach within decontam (v1.22.0) eliminated 71 more contaminant ASVs (Davis et al., 2018). In total, 4,035 unique ASVs were identified as contaminants and removed, resulting in 18,969 unique ASVs and a final median read count of 105,646 per sample for downstream analysis.

#### Diversity analysis

A phylogenetic tree was constructed from the decontaminated ASV sequences using MAFFT and FastTree within the QIIME2 environment (*qiime phylogeny align-to-tree-mafft-fasttree)* to use for phylogenetic-based diversity analysis (Katoh and Standley, 2013; Price et al., 2010). Using the R phyloseq package (v1.46.0), samples were then subjected to repeated rarefaction (n = 100) without replacement to a depth of 13,074 counts (the minimum library size among all samples) for alpha diversity comparisons (*rarefy_even_depth*) (McMurdie and Holmes, 2013). For beta diversity comparisons, raw count data were normalized to relative abundances. Beta diversity indices were then computed using Bray-Curtis and Jaccard dissimilarities (via the vegan v2.6–6.1 R package (Oksanen et al., 2024)) and weighted and unweighted UniFrac distances (via phyloseq v1.46.0). The resulting dissimilarity matrices were ordinated using nonmetric multidimensional scaling (NMDS). Statistical differences were assessed using PERMANOVA (*adonis2* function from the vegan package). Finally, the top ASVs with abundances correlated to the NMDS coordinates were identified using Spearman correlation.

#### Taxonomic and differential abundance testing analysis

Differential abundance (DA) testing was performed on a filtered ASV feature table (raw counts as input), excluding ASVs present in fewer than 10% of samples or with fewer than 100 counts per sample. This filtering was performed to manage the burden of multiple-test correction and reduce tool-specific variation across multiple DA test methods (Nearing et al., 2022). This resulted in 4,823 ASVs as input. To ensure robust results, multiple methods were employed for DA testing, including Maaslin2 (v1.16.0, normalization = “TSS”, transform = “AST”) (Nearing et al., 2022; Mallick et al., 2021), DESeq2 (v1.42.1, default parameters) (Love et al., 2014), ANCOM-BC2 (v2.4.0, default parameters) (Lin and Peddada, 2024), and ALDEx2 (v1.34.0, default parameters) (Fernandes et al., 2013). ASVs identified as statistically significant (q < 0.05) by at least two methods were considered differentially abundant between age groups. For additional taxonomic comparisons of taxa of interest, the ASV feature table was converted to relative abundances and collapsed to the specified taxonomic level (species, genus, phylum). Statistical comparisons between age groups were conducted using the non-parametric Wilcoxon rank sum test. Hierarchical clustering (method = “ward.D2″) was performed on the Bray-Curtis dissimilarity matrix to explore the relationship between age and microbiome composition. Microbiome types based on microbiome composition at the ASV level were identified using Dirichlet multinomial mixtures (DMM), as implemented in the *DirichletMultinomial* R package (v1.44.0) (Holmes et al., 2012). The ASV feature table was first filtered to exclude ASVs present in <30% of samples and those with <100 counts. This stringent filter was applied to eliminate noise from rare taxa and focus on the core, defining members of the community. The lowest Laplace approximation was used to calculate model fit and determine the optimal number of microbiome types per skin site.

#### SPIEC-EASI microbial network inference

The statistical method SPIEC-EASI, implemented in the SpiecEasi R package (v1.0.7) (Kurtz et al., 2015), was used to identify associations between microbial species within each skin site and age group. Briefly, the ASV feature table was collapsed to the species level, retaining ASVs without species-level assignment to minimize data loss. Within each skin site, features present in more than 30% of samples were included in the network analysis, resulting in 194 features for the face (148, 42, and 4 assigned at the species, genus, or family level, respectively) and 144 features for the arm (133, 25, and 6 assigned at the species, genus, or family level, respectively). This filtering step was performed to reduce noise and computational burden, as including too many rare ASVs can yield uninterpretable networks. This prevalence-based approach is consistent with other studies employing SPIEC-EASI (Kurtz et al., 2015; Tipton et al., 2018; Swaney et al., 2022). SPIEC-EASI (method = “mb”, lambda.min.ratio = 1e-2, nlambda = 20) was run on samples grouped by skin site and age group, generating two networks (younger and older age) for each skin site. Network visualization and calculation of topological properties were conducted using the R package igraph (v2.0.3) (Csardi and Nepusz, 2005).

#### Microbial function prediction

To predict microbial community function, the standalone version of PICRUSt2 (v2.5.2) (Douglas et al., 2020) was used to generate MetaCyc pathway abundances (Caspi et al., 2014). Predicted pathways were normalized by relative abundance, followed by calculation of Bray-Curtis dissimilarities and ordination using NMDS. Statistically significant differences were computed using PERMANOVA (*vegan adonis2*). Differential abundance testing of the pathways by age group and skin site was performed using multiple DA methods to ensure robustness of the identified pathways. The following methods were used from the ggpicrust2 R package (v1.7.3) using default parameters: DESeq2, edgeR (Robinson et al., 2010), Maaslin2, limma voom (Ritchie et al., 2015), metagenomeSeq (Paulson et al., 2013), and LinDA (Zhou et al., 2022). Of the methods that produced significant results, only the pathways that were identified by all methods were considered differentially abundant.

## Data Availability

The datasets presented in this study can be found in online repositories. The names of the repository/repositories and accession number(s) can be found below: https://www.ncbi.nlm.nih.gov/, PRJNA1273048.

## Supplementary material

SUPPLEMENTARY FIGURE S1 Alpha and beta diversity age group and skin site comparisons by gender. Alpha diversity metrics were calculated and compared between the old age (OA) group and young age (YA) group for the face and arm, split by gender. Calculations were performed on the ASV feature table and include: **(A)** Observed ASVs, **(B)** Pielou evenness, and **(C)** Shannon diversity. YA (blue) represents the young age group, and OA (red) represents the old age group. Points represent data outliers. Statistical significance for alpha diversity metrics was calculated using the Wilcoxon rank sum test (* *p*-value <0.05). NMDS ordination of the Bray-Curtis dissimilarity matrix for the face of **(D)** female and **(E)** male participants and for the arm of **(F)** female and **(G)** male participants. Statistical significance for compositional differences between age groups was calculated using PERMANOVA, with the *p*-value indicated within each ordination plot. Ellipses represent a 95% confidence interval for each age group. Blue circles represent the young age (YA) group and red triangles represent the old age (OA) group.

SUPPLEMENTARY FIGURE S2 Weighted UniFrac and Bray-Curtis NMDS ordination of face and arm samples. NMDS ordination of the weighted UniFrac distance matrix for the **(A)** face and **(B)** arm. **(C)** NMDS ordination of the Bray-Curtis dissimilarity matrix for all samples. Statistical significance for composition differences was calculated using PERMANOVA, with the *p*-value indicated within each ordination plot. Ellipses represent a 95% confidence interval for each age group. One old age sample from the face was excluded from the weighted UniFrac calculation due to a *Pseudomonas*-dominant microbial community profile that highly skewed the ordination.

SUPPLEMENTARY FIGURE S3 Relative abundances of the top four phyla present by skin site and age group. Phyla not included within the top four phyla are grouped into “Other”. Statistical significance was calculated using the Wilcoxon rank sum test (* *p*-value <0.05). YA = young age group, OA = old age group.

SUPPLEMENTARY FIGURE S4 Genus- and species-level variation of abundant skin taxa between gender and age groups. **(A)** Relative abundance differences between *Cutibacterium, Staphylococcus*, and *Corynebacterium* within each age group, skin site, and gender. Relative abundance differences for species of interest for the **(B)** face and **(C)** arm, split by gender and age group. Statistical significance was calculated using the Wilcoxon rank sum test * *p*-value <0.05, ** *p*-value <0.01. YA = young age group, OA = old age group.

SUPPLEMENTARY FIGURE S5 Differentially abundant taxa between skin sites and age groups. Shown are all differentially abundant ASVs identified for the **(A)** face and **(B)** arm across age groups that were identified by at least two differential abundance testing methods. The horizontal line indicates the mean relative abundance value of each ASV. Multiple ASVs assigned to the same taxon are distinguished with sequential numbering starting from 1. YA = young age group, OA = old age group.

SUPPLEMENTARY FIGURE S6 Top driving ASVs of DMM community types. ASVs identified as the top drivers of Dirichlet multinomial mixtures (DMM) community types are indicated for **(A)** face community type 1, **(B)** face community type 2, **(C)** arm community type 1, **(D)** arm community type 2, and **(E)** arm community type 3. The top drivers are defined as the top 5% of ASVs that contribute the highest absolute weight within each model of community type.

SUPPLEMENTARY FIGURE S7 Age group distribution of Dirichlet multinomial mixtures (DMM) cluster profiles by gender. Number of samples that are assigned to each community type in Figure 5, colored by age group and split by gender, for the **(A)** face and **(B)** arm. YA (blue) = young age, OA (red) = old age.
