# Supplementary figures and images for: Aging-dependent skin microbiome alterations across body sites in a United Kingdom cohort
Source: Front Aging. 2025 Sep 19;6:1644012. doi: 10.3389/fragi.2025.1644012 (PMC12491275; doi:10.3389/fragi.2025.1644012)

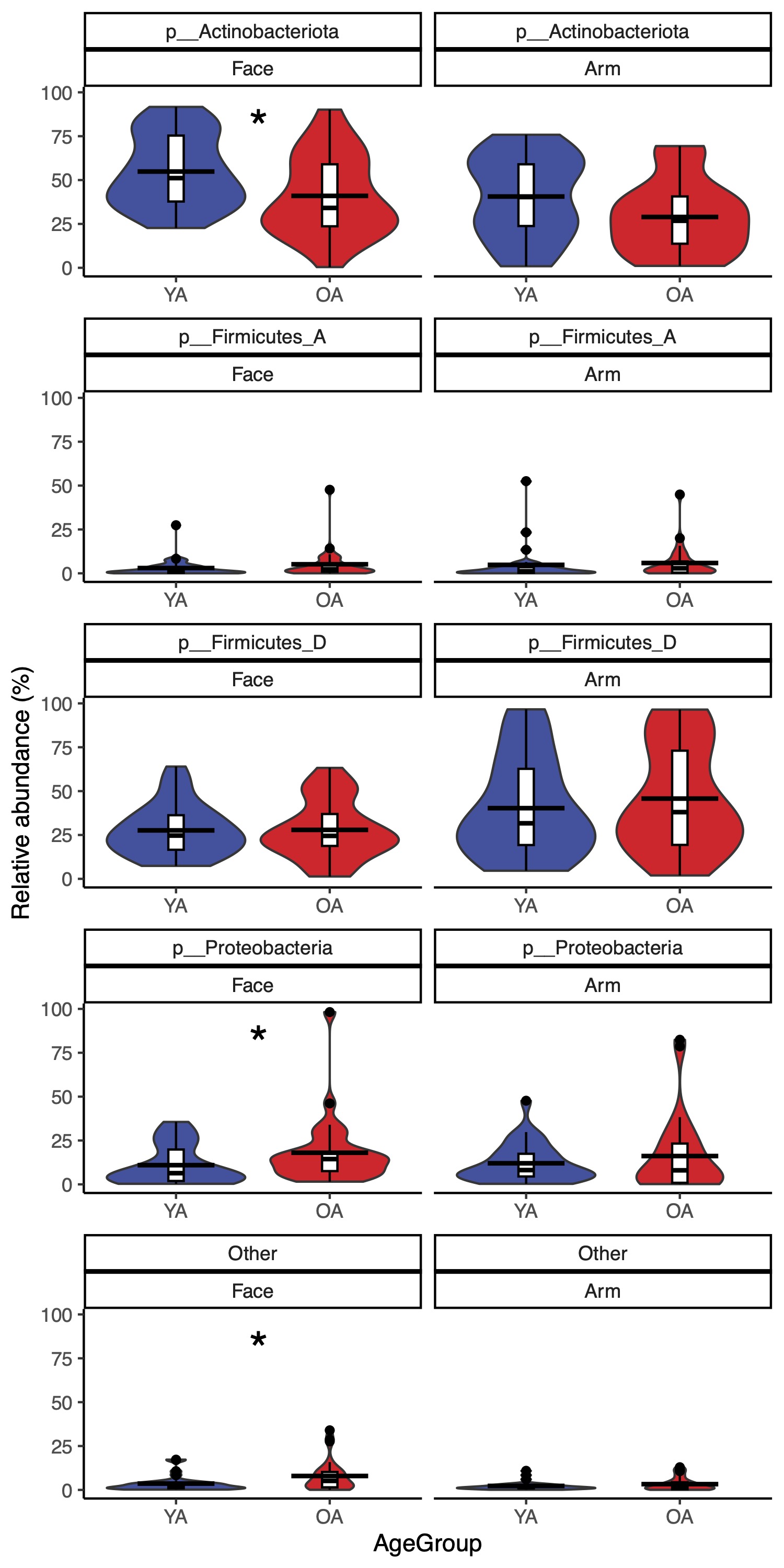

Supplement: Supplementary file 1 [file Image3.jpeg]

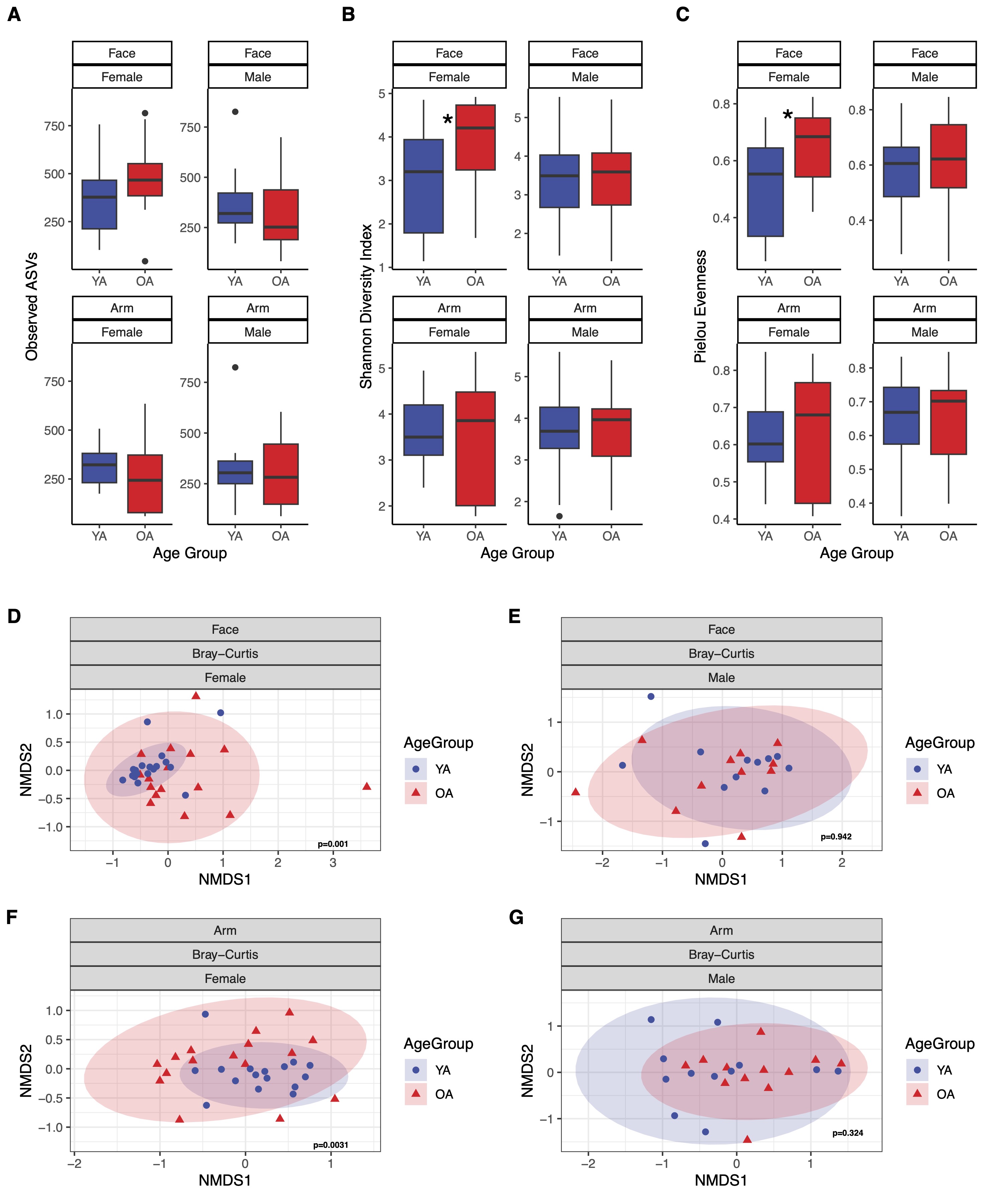

Supplement: Supplementary file 3 [file Image1.jpeg]

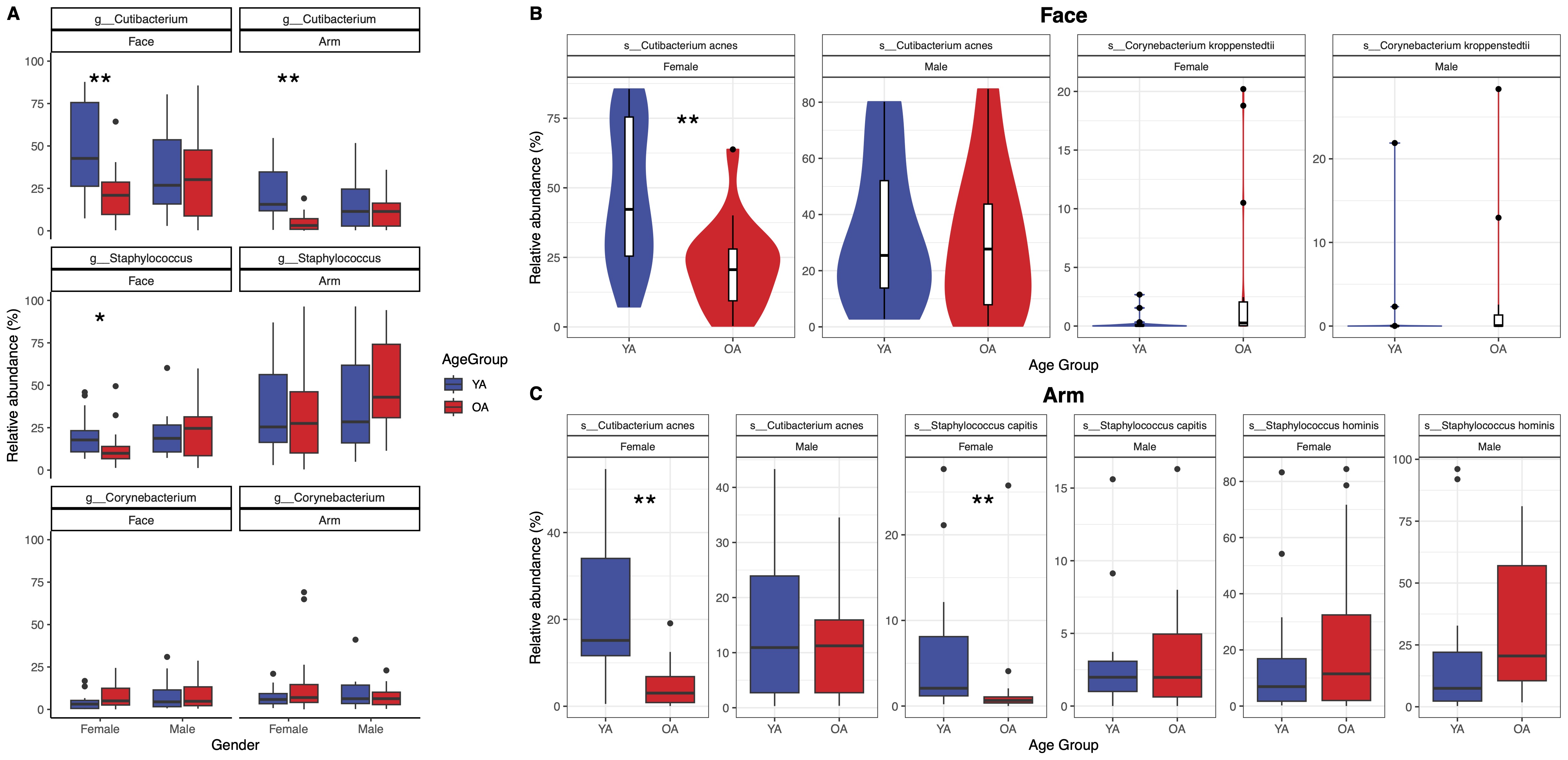

Supplement: Supplementary file 4 [file Image4.jpeg]

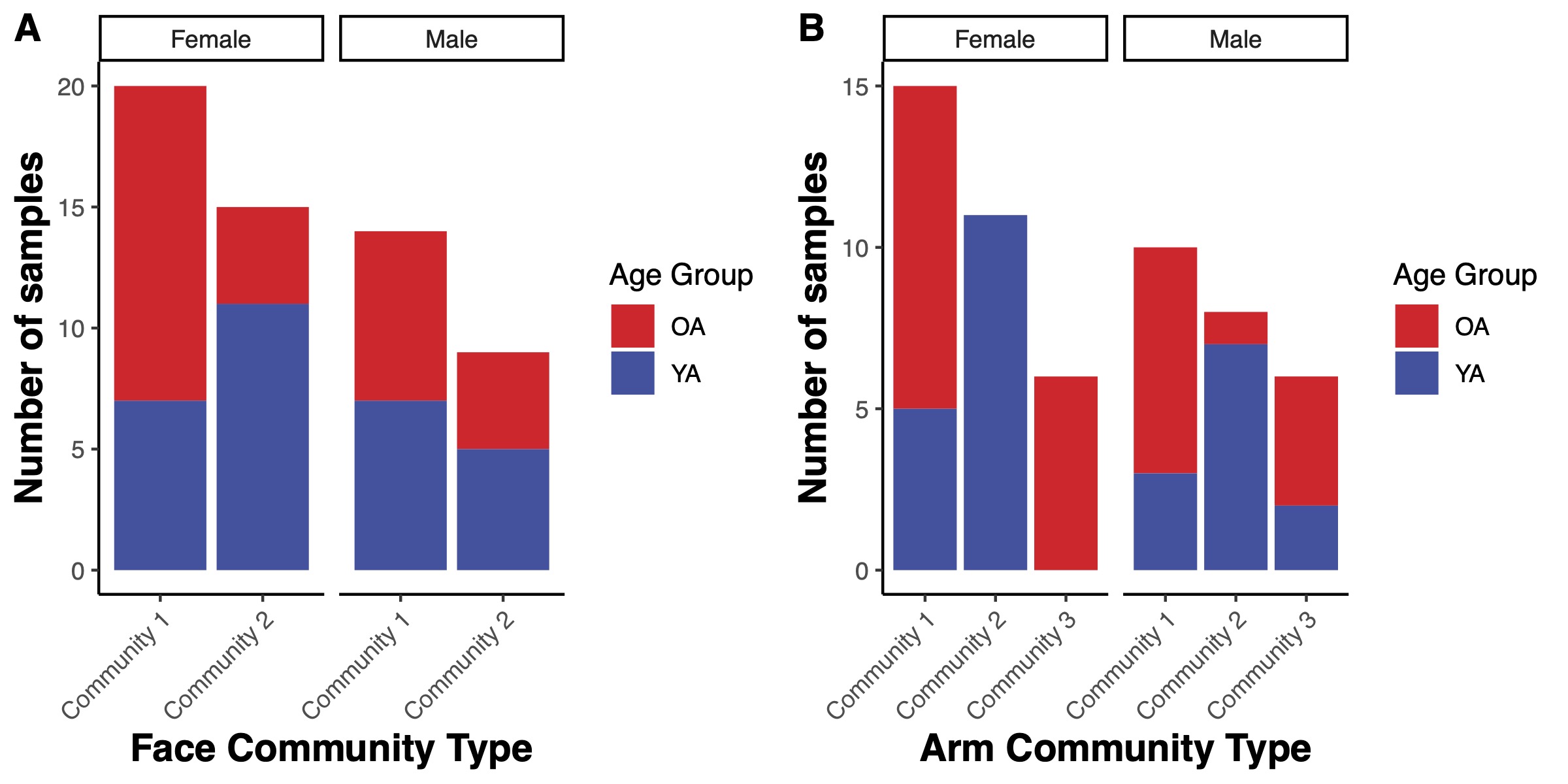

Supplement: Supplementary file 5 [file Image7.jpeg]

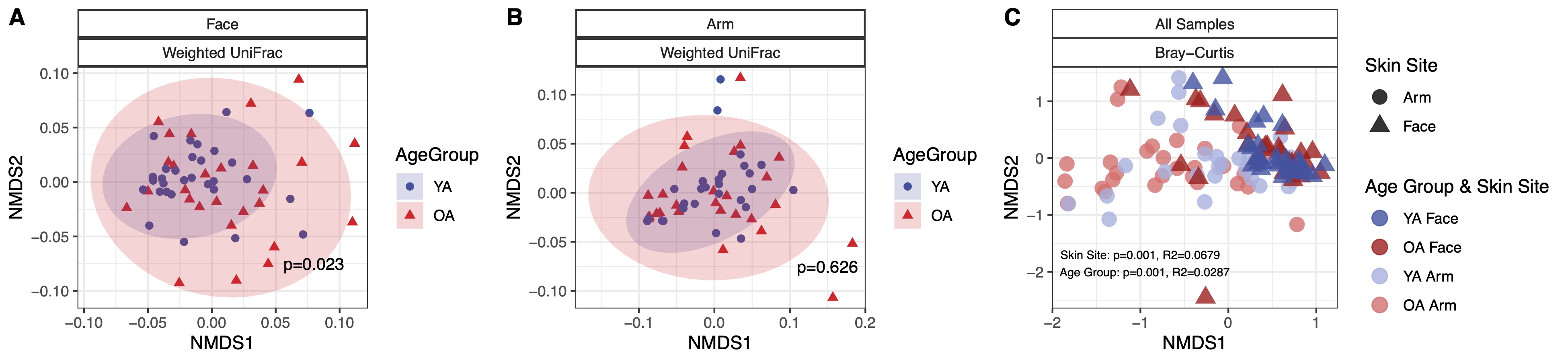

Supplement: Supplementary file 6 [file Image2.jpeg]

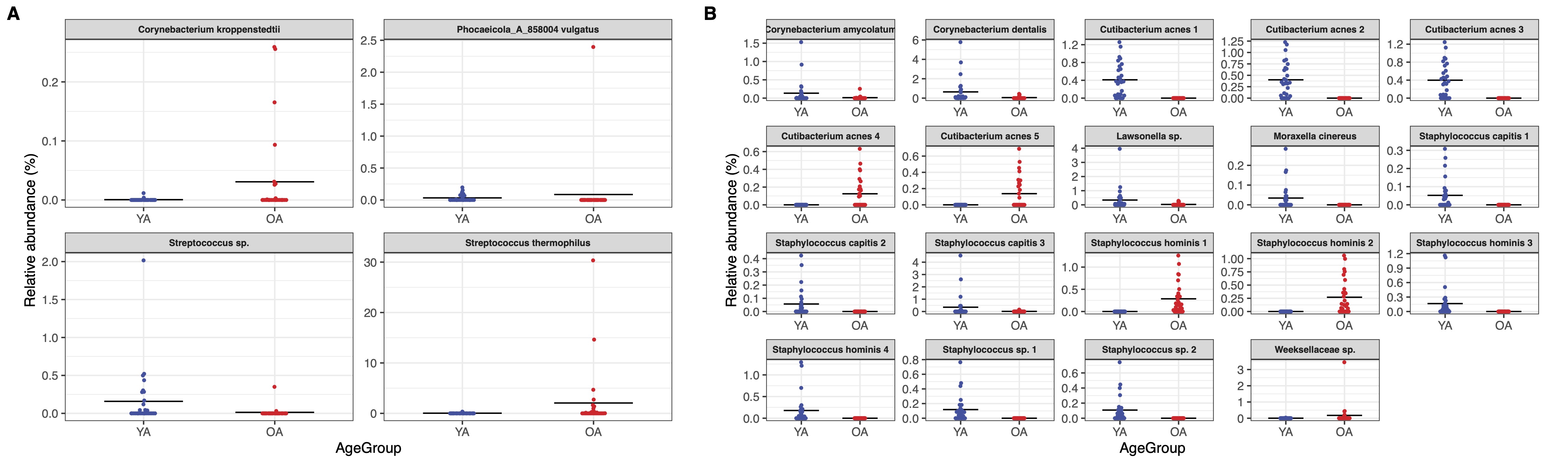

Supplement: Supplementary file 7 [file Image5.jpeg]

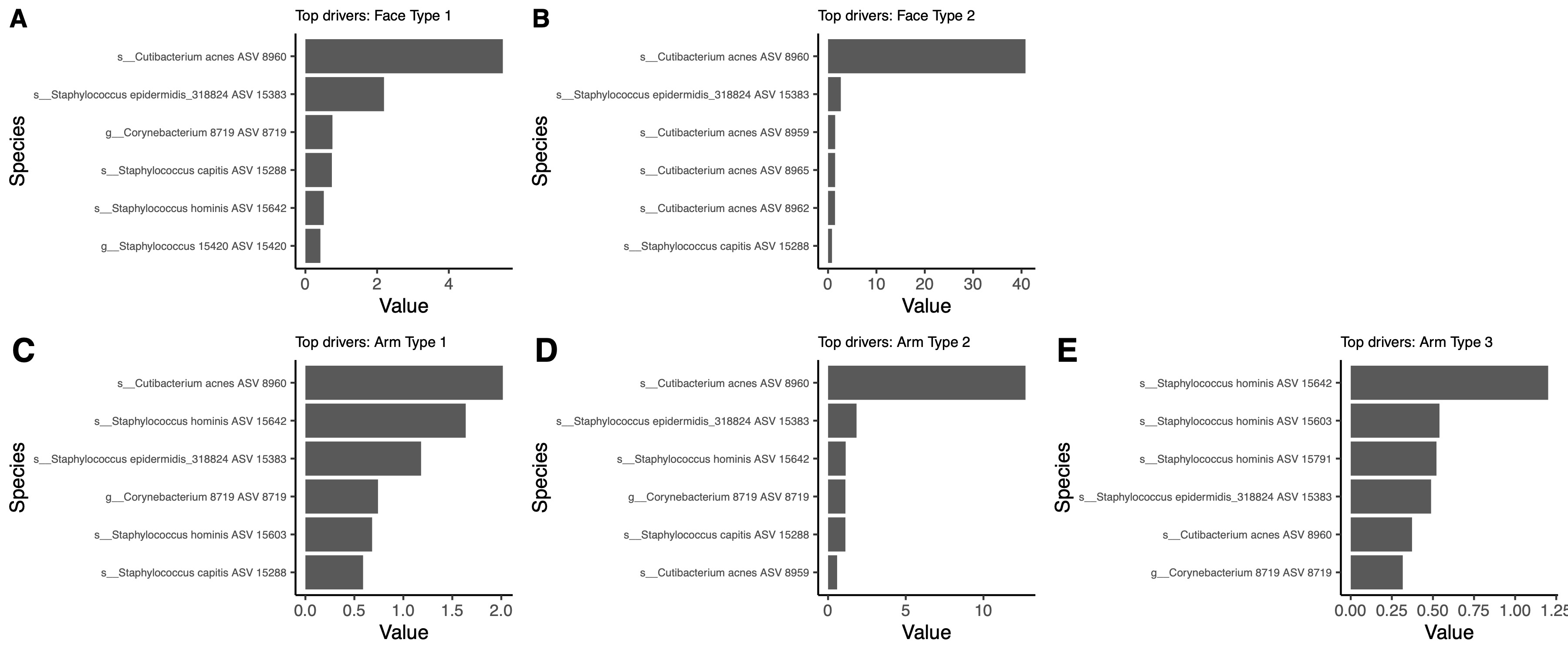

Supplement: Supplementary file 8 [file Image6.jpeg]
