# Supplementary material for: Aging-dependent skin microbiome alterations across body sites in a United Kingdom cohort
Source: Front Aging. 2025 Sep 19;6:1644012. doi: 10.3389/fragi.2025.1644012 (PMC12491275; doi:10.3389/fragi.2025.1644012)
Supplement: Supplementary file 2 [file Table1.docx]

| **Characteristic** | | **Young Adults (n=30)** | **Older Adults (n=29)** | **p-value** |
| --- | --- | --- | --- | --- |
| ***Demographics*** | | | | |
| Age (years), mean (SD) | | 26.7 (4.45) | 72.3 (4.04) | <2.2E-16 |
| Gender, n (%) | | | | 1 |
| *Male* |  | 12 (40%) | 12 (41.4%) |  |
| *Female* |  | 18 (60%) | 17 (58.6%) |  |
| Ethnicity, n (%) | | | | 0.001* |
| *Caucasian* |  | 19 (63.3%) | 29 (100%) |  |
| *Asian* |  | 8 (26.7%) | 0 (0%) |  |
| *Multiple* |  | 1 (3.3%) | 0 (0%) |  |
| *Other* |  | 2 (6.7%) | 0 (0%) |  |
| ***Clinical Characteristics*** | | | | |
| Cardiovascular disease |  | 0 (0%) | 9 (31%) | 0.0008 |
| Type II diabetes |  | 1 (3.3%) | 5 (17.2%) | 0.103 |
| Undergoing hormone treatment,  female n (%) |  | 1 (3.3%) | 4 (13.8%) | 0.195 |
| Immune suppressed, n (%) |  | 1 (3.3%) | 1 (3.4%) | 1 |
| Antibiotic usage**, n (%) |  | 2 (6.7%) | 1 (3.4%) | 0.612 |
| Skin condition, n (%) | | | |  |
| *Acne* |  | 2 (6.7%) | 0 (0%) | 0.492 |
| *Eczema* |  | 4 (13.3%) | 0 (0%) | 0.112 |
| *Psoriasis* |  | 2 (6.7%) | 0 (0%) | 0.492 |

**Supplemental Table 1. Participant Demographic and Self-Reported Clinical Characteristics by Age Group.**

Note: Data are presented as mean ± standard deviation (SD) for continuous variables or as number (n) and percentage (%) for categorical variables. Differences between the Young Adults (n=30) and Older Adults (n=29) were tested as follows: Categorical variables were tested using the Chi-squared (χ2) test or Fisher’s Exact Test (when n<5 in any cell) and continuous variables were tested using the independent samples t-test. A p-value of <0.05 is considered statistically significant.

* Caucasian vs Non-Caucasian

** Current or within the 6 weeks before sampling
